# Plasma phospholipid EPA and DHA are divergently associated with overall mortality in newly diagnosed diabetic patients: results from a follow-up of the Nord-Trøndelag Health (HUNT) Study, Norway

**DOI:** 10.1017/jns.2013.30

**Published:** 2013-11-19

**Authors:** Morten Lindberg, Arne Åsberg, Kristian Midthjell, Kristian S. Bjerve

**Affiliations:** 1 Central Laboratory, Vestfold Hospital Trust, Tønsberg, Norway; 2 Department of Laboratory Medicine, Children's and Women's Health, Norwegian University of Science and Technology, Trondheim, Norway; 3 Department of Medical Biochemistry, St Olavs Hospital, Trondheim University Hospital, Trondheim, Norway; 4 HUNT Research Centre, Department of Public Health and General Practice, Norwegian University of Science and Technology, Trondheim, Norway

**Keywords:** Overall mortality, *n*-3 Fatty acids, *n*-6 Fatty acids, Type 2 diabetes, FADS, fatty acid desaturase, HR, hazard ratio, HUNT, Nord-Trøndelag Health, PLN3, phospholipid *n*-3

## Abstract

Data concerning the long-term effects of *n*-3 and *n*-6 PUFA on disease control and development of complications in diabetic patients are inconsistent. The relationship between plasma phospholipid PUFA and total mortality in type 2 diabetes is unknown. The present study aims to investigate the association between plasma phospholipid fatty acid relative concentrations expressed as weight percentage and total mortality in patients with type 2 diabetes. Mortality rates were evaluated at 5, 10, 15 and 20 years in patients with newly diagnosed diabetes (*n* 323) and matched non-diabetic controls (*n* 200) recruited from the Nord-Trøndelag Health (HUNT) Study, Norway. Kaplan–Meier survival curves were constructed and Cox regression analysis was used to calculate hazard ratios (HR) adjusted for biochemical and clinical covariates. After 10 years of follow-up, EPA in the diabetic population was negatively associated with total mortality, with an HR at the fifth quintile of 0·47 (95 % CI 0·25, 0·90) compared with the first quintile. In contrast, DHA was positively associated with total mortality, with an HR at the fifth quintile of 2·87 (95 % CI 1·45, 5·66). Neither EPA nor DHA was associated with total mortality in matched non-diabetic controls. In conclusion, plasma phospholipid relative concentrations of EPA were negatively associated, while those of DHA were positively associated with total mortality in diabetics. This difference in associations suggests a differential effect of EPA and DHA in patients with type 2 diabetes.

The incidence of type 2 diabetes is increasing in parallel with the obesity epidemic. It is accompanied by medical complications such as dyslipaemia, atherosclerosis, hypertension and a higher risk of death^(^^1^^)^. Therefore, lifestyle factors that may influence type 2 diabetes are of great interest to public health. Long-chain *n*-3 fatty acids are one of many such components that may influence the incidence and course of diabetes. These fatty acids have been shown to mediate beneficial effects on CVD in several populations^(^^2^^–^^5^^)^. However, data are inconsistent regarding the effects of long-chain *n*-3 fatty acids on glucose metabolism and the risk of diabetes. In individuals with type 2 diabetes, a high intake of fish oil moderately increased blood glucose and decreased insulin sensitivity in a randomised control trial^(^^6^^)^ while a cross-over study showed impairment of glycaemic control with a diet rich in polyunsaturated fat^(^^7^^)^. Using FFQ, observational studies have shown an inverse relationship between fish intake and incidence of diabetes and glucose intolerance^(^^8^^,^^9^^)^. Other investigators have reported an increased risk of type 2 diabetes^(^^10^^–^^12^^)^. It is unclear whether these associations are mediated through long-chain *n*-3 fatty acids or other components of fish (i.e. Se, Hg). In meta-analyses, fish oil supplementation has been shown to be associated with no increase^(^^13^^)^ or a non-significant increase^(^^14^^,^^15^^)^ in fasting blood glucose and HbA1c. Data on the association between long-chain *n*-3 fatty acids and mortality in patients with diabetes are limited^(^^16^^–^^18^^)^. The association between the relative concentrations of individual plasma phospholipid long-chain *n*-3 and *n*-6 fatty acids and total mortality in patients with type 2 diabetes is unknown. We investigated this relationship in patients and matched controls.

## Subjects and methods

### Subjects

The present study started as a part of the Nord-Trøndelag Health (HUNT) Study and the HUNT1 survey that was conducted during 1984–1986 in the county of Nord-Trøndelag, Norway^(^^19^^)^. The county is fairly representative of the whole of Norway, ethnically homogeneous with 97 % of Caucasian origin. All inhabitants aged 20 years and older were invited to the survey, and 74 977 (88·1 %) attended the baseline screening. Participants aged 40 years and older had a random, non-fasting capillary blood glucose measured. If the glucose concentration was ≥8 mmol/l in individuals without known diabetes, an appointment was made for fasting blood glucose and, if necessary, an oral glucose tolerance test. Using the 1980 WHO criteria^(^^20^^)^ for diabetes mellitus, a total of 428 new cases of diabetes were identified. From this population of patients with newly confirmed diabetes, 323 subjects were recruited to participate in a prospective study. The study population has been described in detail elsewhere^(^^21^^)^. The present study reports the association between plasma phospholipid fatty acid relative concentrations expressed as weight percentage and total mortality in this population. We also performed identical analyses in a group of 200 healthy controls recruited from the HUNT1 population, matched to patients by sex, age and municipality of residence. The present study was conducted according to the guidelines laid down in the Declaration of Helsinki and all procedures involving human subjects/patients were approved by the Norwegian Data Inspectorate which also considered the legal issues of the study. Written informed consent was obtained from all subjects. Patients and controls were followed until death or 31 December 2006, whatever came first. Death certificates were obtained from the Death Registry at Statistics Norway, which receives the death certificates of all Norwegian citizens. Data on current smoking status, CVD, physical activity and education were obtained from questionnaires.

### Method

Weight and height were measured by trained staff at the screening site. BMI was calculated as kg/m^2^. Blood pressure was measured using a calibrated Hg manometer and the mean of the two measurements was used. Hypertension was defined as systolic blood pressure ≥140 and diastolic blood pressure ≥90 mmHg or current use of antihypertensive drugs. Serum total cholesterol was measured with reagents from Boehringer Mannheim using a Hitachi 737 analyser (Boehringer Mannheim GmbH). HDL-cholesterol was measured with the same method after precipitation with heparin/Mn. Serum creatinine was measured using a modified Jaffe's method on a Kone Diagnostics instrument (Konelab Corp.). The creatinine values were recalibrated to be traceable to an isotope-dilution MS method, and the glomerular filtration rate was estimated with the four-variable formula from the Modification of Diet in Renal Disease Study^(^^22^^)^. Glycosylated Hb (total HbA1) was measured using an agar electrophoresis method with a Corning GLYTRAC™ glycosylated Hb set (Corning Medical). Non-fasting capillary blood glucose and glucose readings in the glucose tolerance test were measured using the Reflocheck method (Boehringer Mannheim GmbH). Timed overnight urine samples were collected for the determination of urinary albumin excretion rate. Urine albumin was measured on a Cobas Fara (Roche Diagnostics Ltd) analyser using reagents from Dako. The fasting plasma samples were prepared and stored at –80°C until analysis. Plasma lipids were extracted with butanol^(^^23^^)^ and phospholipids isolated by column chromatography after adding diheptadecanoyl-glycerophosphocholine and butylated hydroxytoluene (Sigma Chemical) as an internal standard and antioxidant, respectively. The phospholipids were transmethylated and fatty acids quantified by GLC on a Hewlett-Packard 5890A using a 30 m SP2330 fused silica capillary column, 0·25 mm internal diameter, 0·20 µm film thickness (Supelco Inc.) essentially as previously described^(^^24^^)^. The results were expressed as mg of phospholipid fatty acids per litre plasma and recalculated to a percentage by weight on the basis of twenty-two identified fatty acids. A normal human serum sample was included in each run to monitor analytical performance. The between-series CV for the complete study period (*n* 83) were 3·7, 3·7 and 4·9 %, respectively, for 20 : 4*n*-6, 20 : 5*n*-3 and 22 : 6*n*-3 when measured as mg/l. The corresponding CV were reduced to 2·2, 2·4 and 3·5 % when concentrations were expressed as weight percentage.

### Statistical analysis

Baseline characteristics are reported as mean values and standard deviations or proportions as appropriate. Reported *P* values are two-sided, and CI are computed at the 95 % level. The Wilcoxon rank sum test was used to compare continuous data from patients and matched controls. Survival analysis was performed separately in the diabetic population and matched controls. Cox proportional hazard regression analysis was used to estimate risk, with censoring at death or specified time of follow-up. Hazard ratios (HR) at increasing quintiles were calculated from the regression equation using the quintile median of the respective fatty acids. According to the research protocol, EPA, DHA and the phospholipid *n*-3 (PLN3) index^(^^25^^,^^26^^)^ were included in the analysis. To investigate the significance of other fatty acids and desaturase enzyme indexes, we performed a stepwise selection including all PUFA with a carbon chain ≥18 carbon atoms and the fatty acid ratios 20 : 3*n*-6/18 : 2*n*-6 and 20 : 4*n*-6/20 : 3*n*-6 as indexes of Δ-6 desaturase and Δ-5 desaturase, respectively. Using the criteria *P* = 0·25 for entering and *P* = 0·05 for staying in the model, 22 : 4*n*-6 (adrenic acid) was included in the model. We used fractional polynomials to investigate possible non-linear functional relationships^(^^27^^)^. The fractional polynomial fitting algorithm converged after three cycles. In addition to adrenic acid from the stepwise selection, the multivariate model was adjusted for major risk factors of death in the general population (age, sex, BMI, total cholesterol, HbA1c, mean blood pressure, education, exercise, current smoking and estimated glomerular filtration rate). EPA and DHA were analysed with and without mutual adjustment. Multicollinearity was not present as assessed by the variance inflation factor. The proportional hazard assumption was confirmed using Schoenfeld residuals. Statistical computations were performed using SAS/STAT software version 9.2 (SAS Institute Inc.) and STATA software version 12 (StataCorp LP).

## Results

Baseline characteristics for 323 patients and 200 matched controls are given in Table 1. The mean age of patients with diabetes was 68·2 (sd 9·8) years. Patients with diabetes had significantly higher BMI, less physical activity, higher mean blood pressure and were more prone to CVD compared with controls (*P* < 0·001, *P* = 0·004, *P* < 0·001 and *P* = 0·002, respectively). Baseline plasma phospholipid fatty acid relative concentrations expressed as weight percentage are given in Table 2. In patients with diabetes, SFA accounted for 42 % of total fatty acids, MUFA for 14 %, *n*-6 PUFA for 33 % and *n*-3 PUFA for 11 %, leading to an *n*-6:*n*-3 ratio of 3·0. The corresponding values in controls were not statistically different. Likewise, no statistically significant differences were found for EPA (*P* = 0·26) or DHA (*P* = 0·19).

**Table 1.** Baseline characteristics of the study participants (Mean values and standard deviations, number of subjects and percentages)

	Diabetics		Controls*	
	Mean	sd	Mean	sd
Subjects (*n*)	323		200	
Age (years)	68·2	9·8	66·6	9·8
Women				
*n*	156		97	
%	48		49	
BMI (kg/m^2^)	29·3	4·85	26·2	3·50
Total cholesterol (mmol/l)	6·7	1·3	7·3	1·5
HDL-cholesterol (mmol/l)	1·2	0·3	1·5	0·3
eGFR (ml/min per 1·73 m^2^)	98	30	91	20
HbA1 (%)	6·9	1·3	6·0	0·6
Albumin excretion rate (µg/min)	27·5	66·0	14·6	18·6
Current smoker				
*n*	105		65	
%	33		33	
CVD†				
*n*	80		27	
%	25		14	
Mean blood pressure (mmHg)	126	17	119	17
Hypertension‡				
*n*	216		87	
%	67		44	
Higher education§				
*n*	13		12	
%	4		6	
Weekly exercise				
*n*	139		112	
%	43		56	

eGFR, estimated glomerular filtration rate;

* Healthy controls matched by sex, age and residence.

† Known angina, stroke or myocardial infarction.

‡ Systolic blood pressure ≥140 mmHg and diastolic blood pressure ≥90 mmHg or antihypertensive medication.

§ Completed ≥12 years education.

**Table 2.** Baseline plasma phospholipid fatty acid relative concentrations (weight percentage; wt %) (Mean values and standard deviations)

	Individuals with diabetes (*n* 323)		Controls* (*n* 200)	
	Mean	sd	Mean	sd
Fatty acid (wt %)				
14 : 0	0·32	0·08	0·32	0·09
16 : 0	24·2	1·52	23·7	1·51
18 : 0	13·2	0·95	13·4	0·95
20 : 0	0·65	0·13	0·66	0·13
22 : 0	2·33	0·43	2·42	0·54
24 : 0	0·97	0·25	0·99	0·24
16 : 1	0·52	0·17	0·52	0·22
18 : 1	10·9	1·24	10·8	1·30
20 : 1	0·32	0·12	0·35	0·11
22 : 1	0·40	0·18	0·40	0·19
24 : 1	1·85	0·54	1·90	0·51
20 : 3*n*-9	0·17	0·09	0·15	0·10
18 : 3*n*-3	0·22	0·11	0·24	0·09
20 : 5*n*-3	2·14	1·20	2·10	1·28
22 : 5*n*-3	1·28	0·22	1·32	0·22
22 : 6*n*-3	7·11	1·60	7·35	1·87
18 : 2*n*-6	20·0	2·92	20·8	2·82
20 : 2*n*-6	0·46	0·09	0·47	0·12
20 : 3*n*-6	3·04	0·68	2·74	0·73
20 : 4*n*-6	8·79	1·29	8·36	1·20
22 : 4*n*-6	0·58	0·18	0·61	0·19
22 : 5*n*-6	0·12	0·05	0·12	0·05
PLN3 index†	9·25	2·55	9·46	2·90
Sum NEFA (mg/l)	1335·3	210·5	1377·9	201·5

PLN3, phospholipid *n*-3.

* Controls matched by sex, age and municipality of residence attended a similar examination.

† PLN3 index = EPA + DHA.

**Fig. 1.**
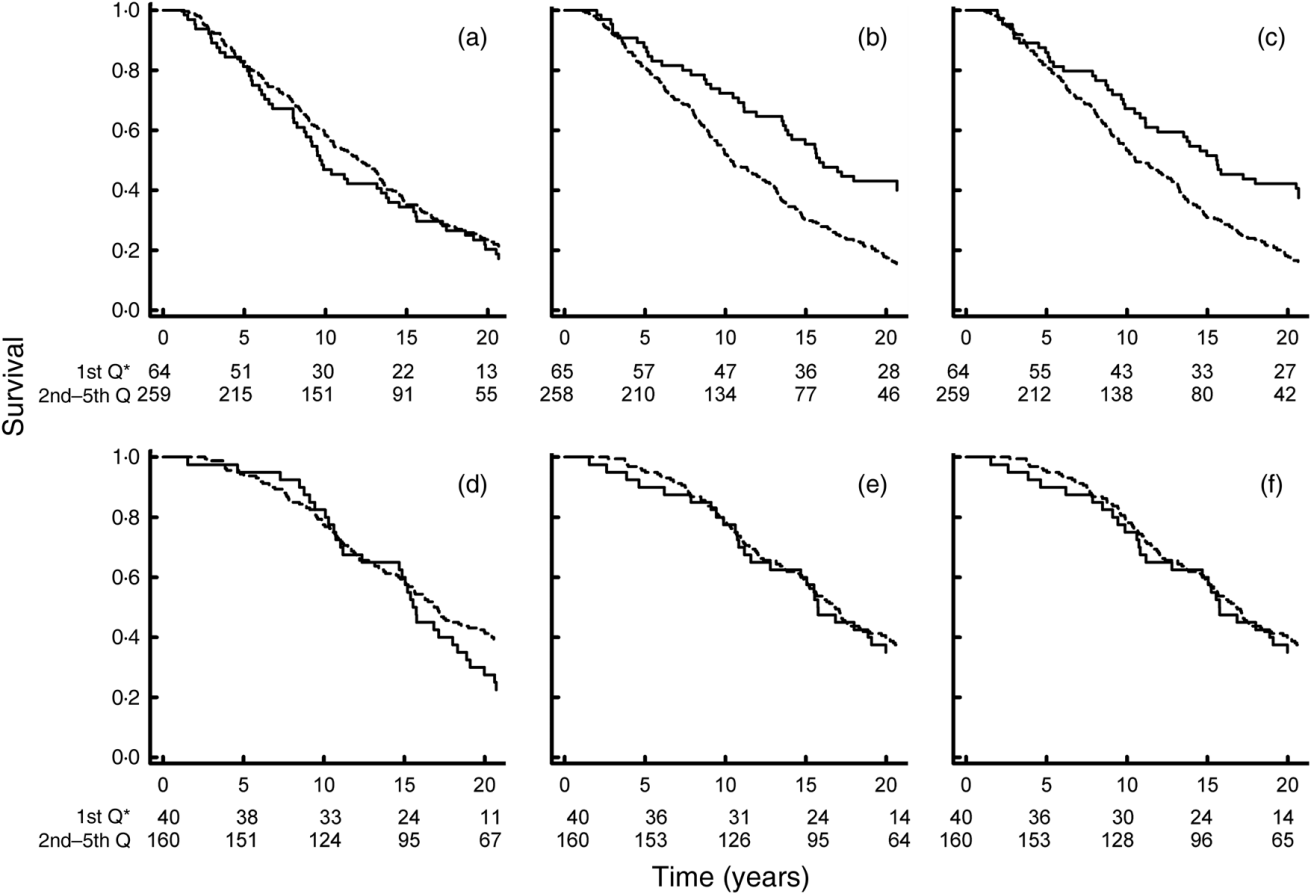
Kaplan–Meier plots of survival in diabetics ((a), (b) and (c)) and matched controls ((d), (e) and (f)) according to quintiles (Q) of EPA ((a) and (d)), DHA ((b) and (e)) and phospholipid *n*-3 (PLN3) index ((c) and (f)). ———, First quintile; ---------, second to fifth quintile. * Numbers at risk in the first quintile and the combined second to fifth quintiles, respectively.

**Fig. 2.**
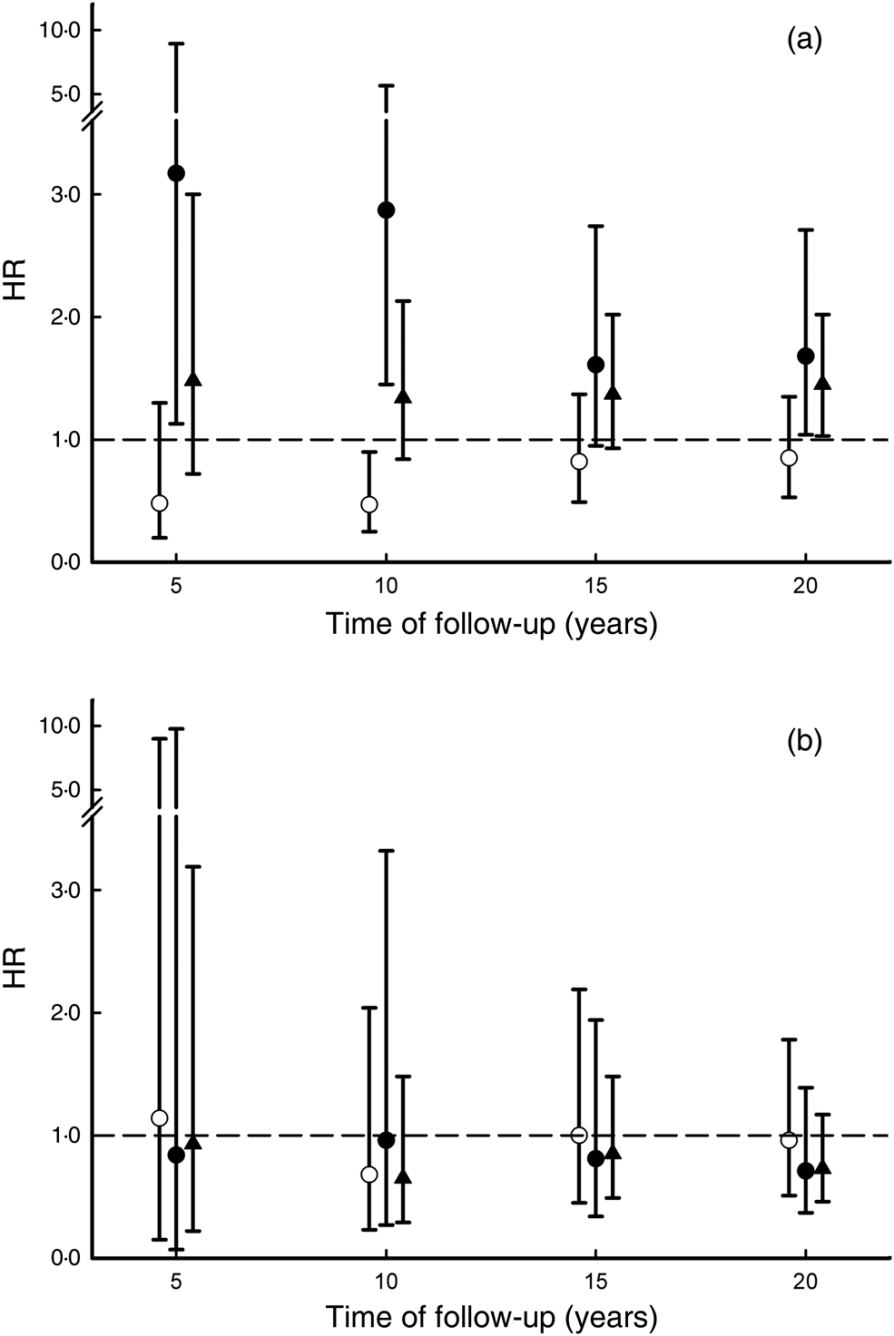
Hazard ratios (HR) and 95 % CI of the fifth *v.* the first quintile of EPA (20 : 5*n*-3; ○), DHA (22 : 6*n*-3; •) and phospholipid *n*-3 (PLN3) index (▴; EPA + DHA) at 5 to 20 years of follow-up. (a) Patients with newly diagnosed diabetes (*n* 323); (b) controls matched by sex, age and municipality of residence (*n* 200).

Kaplan–Meier survival plots corresponding to the lowest and the combined four highest quintiles of EPA, DHA and PLN3 index are shown in Fig. 1. Figs. 1(a–c) depict patients with diabetes, whereas Figs. 1(d–f) represent matched controls. After 20 years of follow-up, patients with diabetes experienced 249 (77 %) deaths whereas the control group experienced 122 (61 %) deaths. In diabetics the mortality rate was higher in the upper four quintiles as compared with the lowest quintile for both DHA and the PLN3 index (log-rank test, *P* < 0·001 and *P* = 0·006, respectively). There were no differences between strata for EPA in patients, or in strata of EPA, DHA or PLN3 index in matched controls. Fig. 2 illustrates the relationship between follow-up time and the HR of the fifth *v.* first quintile for EPA, DHA and PLN3 index in diabetic patients (Fig. 2(a)) and controls (Fig. 2(b)). The shown HR are point estimates calculated from the Cox regression equations for the median relative fatty acid concentration of the first and the fifth quintiles of EPA, DHA and PLN3, respectively. The general pattern of divergent associations between EPA, DHA and total mortality is visible after 5 years of follow-up in diabetic patients. Neither EPA, DHA nor PLN3 index had any statistically significant association with HR in matched controls. With increasing follow-up time, regression dilution bias decreases the strength of the observed associations. Table 3 shows the results from Cox regression analysis of HR for EPA, DHA and PLN3 index, with increasing fatty acid relative concentrations in diabetic patients. When calculating HR as a function of EPA we included DHA in the model, and vice versa, in order to adjust for any potentially different clinical effects. Using the first quintile as reference, we calculated the HR at increasing quintile points. The HR of EPA at the fifth quintile was 0·47 (95 % CI 0·25, 0·90), and the corresponding HR for DHA and PLN3 index were 2·87 (95 % CI 1·45, 5·66) and 1·34 (95 % CI 0·84, 2·13), respectively. Similar results were found when HR was calculated without adjusting for adrenic acid, EPA or DHA. Using the first quintile as reference, the HR of EPA at the fifth quintile was now 0·89 (95 % CI 0·56, 1·44; *P* = 0·645), and the corresponding HR for DHA and PLN3 index were 1·67 (95 % CI 1·02, 2·71; *P* = 0·04) and 1·36 (95 % CI 0·86, 2·16; *P* = 0·193), respectively. There was no statistically significant association between the PLN3 index and overall mortality in the present study.

**Table 3.** Calculated risk of death in 323 diabetic patients at 10 years of follow-up as a function of increasing fatty acid quintiles* (Hazard ratios (HR) and 95 % confidence intervals)

	EPA			DHA			PLN3 index†		
Quintile	Wt %	HR‡§	95 % CI	Wt %	HR‡§	95 % CI	Wt %	HR‡	95 % CI
1	1·10	1·00	Reference	5·17	1·00	Reference	6·58	1·00	Reference
2	1·42	0·85	0·74, 0·98	6·11	1·27	1·09, 1·49	7·54	1·05	0·97, 1·13
3	1·79	0·74	0·57, 0·96	6·86	1·52	1·16, 1·99	8·72	1·11	0·94, 1·30
4	2·36	0·63	0·42, 0·94	7·87	1·95	1·27, 3·00	10·2	1·18	0·91, 1·53
5	3·67	0·47	0·25, 0·90	9·43	2·87	1·45, 5·66	12·8	1·34	0·84, 2·13

PLN3, phospholipid *n*-3; Wt %, weight percentage.

* HR were calculated for each fatty acid using the median relative concentration within each quintile and using the first quintile as reference.

† PLN3 index = EPA + DHA.

‡ Adjusted for age, sex, BMI, total cholesterol, HbA1c, mean blood pressure, education, exercise, current smoking and estimated glomerular filtration rate, and adrenic acid. The Cox model *P* values were: EPA (*P* = 0·023), DHA (*P* = 0·002) and PLN3 index (*P* = 0·223).

§ When calculating HR for EPA and DHA, DHA and EPA, respectively, were included in the adjustment.

## Discussion

In this prospective study of 323 patients with newly diagnosed diabetes, EPA in plasma phospholipids was inversely associated with total mortality after 10 years of follow-up whereas DHA was associated with an increased risk of death. The composite PLN3 index showed a pattern similar to DHA, possibly reflecting that DHA contributes on average 77 % of the index. No significant associations were observed in the control population. The patients in the present study were recruited from a population-based health survey and thereby represent all individuals with newly diagnosed type 2 diabetes in this population. Blood samples for fatty acid measurements were collected at baseline, reflecting metabolic regulation and dietary intake in patients with diabetes before therapeutic intervention. Some study limitations should be considered. Although the variance inflation factor did not indicate severe multicollinearity, the intercorrelation between dietary intakes of EPA and DHA could limit the ability to differentiate independent associations between individual fatty acids and outcome. Another issue is the use of plasma phospholipid fatty acids as a marker of long-term dietary intake. There is currently no consensus as to which material (adipose tissue, serum, plasma, erythrocytes), lipid compartment (phospholipids, cholesteryl esters, total lipids) and fatty acid (EPA, DHA, Omega-3 index) best reflect long-chain marine *n*-3 fatty acid intake. Factors such as availability and stability of samples, handling requirements and time period of interest may influence the choice of preferred marker. We have recently investigated the long-term tracking of plasma phospholid fatty acids and reported correlations similar to other commonly used clinical biochemical markers such as total cholesterol and TAG, supporting their validity as dietary markers in longitudinal studies^(^^21^^)^. Finally, possible misclassification over time and consequently regression dilution bias is a potential study limitation. This might explain the decreasing association between HR and EPA, DHA and PLN3 index seen in diabetic patients (Fig. 2(a)). Norwegian national dietary advice for both diabetic patients and healthy individuals as well as the medical treatment of patients with type 2 diabetes remained largely unchanged during the study period. It seems therefore less likely that dietary or medication changes could confound the present results. For EPA, the Kaplan–Meier curves of the first quintile *v.* the second to fifth quintile did not show a statistically significant difference in patients with diabetes (Fig. 1(a)); however, the curves did indicate a protective effect of EPA at about 10 years of follow-up. This association is more pronounced in the Cox regression analysis where the HR of the fifth *v.* first quintile is calculated, adjusted for biochemical and clinical covariates (Fig. 2(a)).We cannot tell whether the observed difference in associations is due to differential effects of EPA and DHA, differential effects in type 2 diabetes and controls, or a combination of both. The individual effects of EPA and DHA have been reviewed recently^(^^28^^,^^29^^)^. Fatty acids exert their effects through a variety of mechanisms, including transcription factors and signal molecules. Animal studies support an insulin-sensitising action from both EPA and DHA given individually^(^^30^^,^^31^^)^. In human subjects, reports are inconsistent. Mostad *et al.*^(^^6^^)^ showed that a high intake of fish oil moderately increased blood glucose and decreased insulin sensitivity in patients with type 2 diabetes. Another study on patients with type 2 diabetes treated for hypertension reported an increase in fasting glucose, but observed no significant association with glycated Hb, fasting insulin or insulin sensitivity^(^^32^^)^. In healthy human subjects, neither EPA nor DHA affected insulin sensitivity or HbA1c levels while EPA tended to increase glucose levels^(^^33^^)^.

The protective effect of *n*-3 fatty acids on CHD and mortality is well documented^(^^2^^,^^3^^,^^5^^)^. Several mechanisms have been proposed to convey this effect, including reduction of blood TAG^(^^34^^)^, prevention of coronary atherosclerosis^(^^35^^)^ and possibly anti-arrhythmic effects^(^^36^^)^. Diabetes is characterised by abnormalities in the lipid metabolism^(^^37^^)^. Therefore, *n*-3 fatty acids that modify these pathways may reduce the incidence and mortality from CVD in patients with diabetes. To the best of our knowledge, the present study is the first to investigate the relationship between total mortality and individual fatty acids in a newly diagnosed, previously untreated diabetic population. The Heart and Soul study reported an inverse association between baseline blood *n*-3 levels and total mortality in patients with stable CHD. Patients having baseline EPA + DHA levels at or above the median had a 27 % decreased risk of death compared with those below the median (HR 0·73; 95 % CI 0·56, 0·94)^(^^38^^)^. In the Nurses' Health Study, Hu *et al.*^(^^16^^)^ investigated the association between the intake of fish and long-chain *n*-3 fatty acids, calculated from FFQ, and the risk of CHD and total mortality in women with diabetes. Comparing the highest with the lowest quintile, they reported a trend towards a lower incidence of CHD (relative risk (RR) 0·69; 95 % CI 0·47, 1·03) and total mortality (RR 0·63, 95 % CI 0·45, 0·88). Among patients with diabetes included in the GISSI-HF (Gruppo Italiano per lo Studio della Sopravvivenza nell'Infarto Miocardico-Prevenzione-Heart Failure) trial, supplementation with 1 g of *n*-3 PUFA daily (where the EPA:DHA ratio was 1:1·2) reduced the composite endpoint of all-cause mortality or admission to hospital for cardiovascular reasons by 11 % (HR 0·89; 95 % CI 0·80, 0·99)^(^^17^^)^. Due to study design it was not possible to evaluate the association with individual fatty acids in any of these studies.

Mozaffarian *et al.*^(^^39^^)^ recently reported that both EPA and DHA in plasma phospholipids were negatively associated with total mortality in an older U.S. adult population. They also found that estimated years of remaining life gained in the highest compared with the lowest quintile of *n*-3 fatty acids were similar in diabetics and non-diabetics. However, their multivariate model included diabetes only as a confounding variable, assuming that the association between mortality and marine *n*-3 fatty acids is described by the same multivariate function in diabetics and non-diabetics. In contrast, the statistical analysis used in the present report allows for the possibility that the functions describing the association between marine *n*-3 fatty acids and total mortality might be different in diabetics and non-diabetics. The medians of the lowest and highest quintiles of the US population^(^^39^^)^ are 0·30 and 0·92 for EPA, and 1·95 and 4·34 for DHA. The corresponding medians in the Norwegian population are 1·10 and 3·67 for EPA, and 5·17 and 9·43 for DHA (Table 3). Assuming that the methods used in this paper and by Mozaffarian *et al.*^(^^39^^)^ to quantitfy EPA and DHA give comparable results, the highest quintiles in the US population are lower than the lowest quintile in the Norwegian population. This might also contribute to the explanation of why the observed association between total mortality and the relative concentrations of EPA and DHA differs in the two populations.

Fatty acids may affect insulin sensitivity through direct regulatory effects on gene expression and enzyme activity^(^^40^^)^. Strong associations between variants in the human Δ-5 and Δ-6 desaturase genes *FADS1* and *FADS2* (fatty acid desaturase type 1 and type 2, respectively) and fatty acid composition in serum phospholipids have been reported^(^^41^^)^. Recent genetic studies show that polymorphisms in the FADS genes modulate desaturase activity independent of nutritional intake^(^^42^^)^. Using product:precursor ratios, the incidence of type 2 diabetes was directly associated with 18 : 3*n*-6/18 : 2*n*-6 (Δ-6 desaturase activity) and inversely associated with 20 : 4*n*-6/20 : 3*n*-6 (Δ-5 desaturase activity)^(^^43^^)^. Results were corroborated by simultaneous studies of FADS1 and FADS2 genotypes. It is therefore likely that genetic constitution significantly influences the effect of nutrition on complex phenotypes such as type 2 diabetes, inflammation and coronary artery disease. However, in the present study we did not observe any association between product:precursor ratios and overall mortality.

Both EPA and DHA are strongly correlated with the dietary intake of marine food^(^^21^^,^^44^^)^. If EPA and DHA are inversely associated with some clinical effects, the use of a composite variable such as the Omega-3 index could conceal important associations. Clinical studies have suggested that EPA and DHA have divergent clinical effects. Bønaa *et al.*^(^^45^^)^ showed that EPA and DHA were inversely associated with concentration of HDL and apoA-I in a 10-week dietary supplementation trial, and found a strong inverse relationship between the change in HDL-cholesterol and plasma phospholipid DHA (*r* –0·43; *P* = 0·0002), whereas HDL-cholesterol and EPA did not correlate^(^^45^^)^. Martinelli *et al.*^(^^46^^)^, in their study on subjects with and without coronary artery disease, observed significantly higher concentrations of DHA in patients compared with controls (*P* = 0·001), whereas EPA did not differ. Finally, a recent meta-analysis did not find either major harms or benefits of fish/seafood or EPA + DHA on the development of diabetes mellitus^(^^47^^)^.

Our observation that EPA and DHA are divergently accociated with total mortality in diabetic patients suggests that these two fatty acids might have differential effects on total mortality in patients with type 2 diabetes. If these results are confirmed in other studies, it could in part explain the conflicting results regarding long-chain *n*-3 fatty acids and diabetes. It could also lead to a better understanding of essential fatty acid metabolism in diabetes and thereby better dietary advice to the growing population of patients with diabetes.
